# Intravitreal aflibercept for the treatment of patients with neovascular age-related macular degeneration in routine clinical practice in Latin America: the AQUILA study

**DOI:** 10.1186/s40942-022-00425-w

**Published:** 2022-10-18

**Authors:** Lihteh Wu, Arnaldo F. Bordon, Martin Charles, Francisco J. Rodríguez, JinKyung Lee, Tobias Machewitz, Margarete Mueller, Gabriela del Carmen Gay, Jans Fromow-Guerra, the AQUILA investigators

**Affiliations:** 1 Asociados de Macula, Vitreo y Retina de Costa Rica, Primer Piso Torre Mercedes, Calle 24, Paseo Colon, San José 10102 Costa Rica; 2 Hospital Oftalmológico de Sorocaba, R. Nabek Shiroma, 210 – Jarim Emilia, Sorocaba, SP 18031-060 Brazil; 3 Centro Oftalmológico Dr Charles, 841, C116 ABA Riobamba, Buenos Aires Argentina; 4 Fundación Oftalmológíca, Universidad del Rosario School of Medicine, Cl. 50 ##13–50, 110231 Bogotá, Colombia; 5 Bayer AG, 13342 Berlin, Germany; 6 Bayer SA, Doctor Ricardo Gutiérrez 3652, B1605EHD Munro, Buenos Aires Argentina; 7 Macula Retina Consultores, Calle Sur 132, Las Américas, Álvaro Obregón, 01120 Mexico City, Mexico

**Keywords:** Macula, Neovascularization, Vision, Clinical trial

## Abstract

**Background:**

AQUILA (NCT03470103) was a prospective, observational, 12-month cohort study evaluating treatment patterns, clinical effectiveness, and safety of intravitreal aflibercept (IVT-AFL) in patients from Latin America with neovascular age-related macular degeneration (nAMD).

**Methods:**

Treatment-naïve and previously treated (switching to IVT-AFL) patients (aged  ≥ 55 years) were enrolled from March 2018, with a primary completion date of September 2020, from Argentina, Colombia, Costa Rica, and Mexico. Patients received IVT-AFL in a routine clinical practice setting.

**Results:**

Of 274 patients in the full analysis set, 201 were treatment-naïve and 73 had received previous treatment. The mean ± standard deviation number of IVT-AFL injections received by month 12 was 4.2 ± 1.9 (treatment-naïve) and 5.2 ± 2.7 (previously treated). The median duration from diagnosis to IVT-AFL treatment was 1.2 months (treatment-naïve) and 19.5 months (previously treated). Mean best-corrected visual acuity (BCVA) (Early Treatment Diabetic Retinopathy Study [ETDRS] letters) improved from baseline to month 12 by + 5.2 ± 18.3 (treatment-naïve; baseline: 48.2 ± 23.5) and + 3.1 ± 15.3 letters (previously treated; baseline: 47.7 ± 21.4).

**Conclusion:**

AQUILA is the first study to assess the use of IVT-AFL in routine clinical practice in Latin America. Mean BCVA and other visual acuity outcomes improved in both treatment groups, despite many patients not receiving the IVT-AFL label-recommended regimen of three initial monthly doses, or seven or more injections in 12 months. Patients who did receive the label-recommended number of injections had numerically greater improvements in visual acuity outcomes. Patients with nAMD treated regularly and more frequently with IVT-AFL, therefore, have the potential to achieve outcomes consistent with those observed in interventional studies.

*Trial registration:* Clinicaltrials.gov, NCT03470103. Registered February 5, 2018, https://clinicaltrials.gov/ct2/show/NCT03470103

**Supplementary Information:**

The online version contains supplementary material available at 10.1186/s40942-022-00425-w.

## Background

Neovascular age-related macular degeneration (nAMD) is the world’s leading cause of vision loss in adults aged over 65 years [1]. Given that the proportion of Latin Americans predicted to be older than 65 years in 2050 is expected to increase to 18.5%, a corresponding increase in patients with nAMD is also expected and represents a rising concern for health care authorities in Latin America [1].

Anti-vascular endothelial growth factor (anti-VEGF) therapies, such as aflibercept, ranibizumab, and brolucizumab, are approved to treat nAMD (bevacizumab is also used off-label to treat nAMD). These therapies work by inhibiting or blocking VEGF, thus reducing or mediating the growth of abnormal blood vessels, and by reducing leakage of existing blood vessels that may cause vision loss [2, 3]. VIEW 1 and VIEW 2, two similarly designed, prospective, multinational, randomized clinical trials, investigated fixed-dose intravitreal aflibercept (IVT-AFL) treatment for nAMD and described its noninferiority compared to ranibizumab [4]. The success of the VIEW 1 and VIEW 2 trials led to the approval of IVT-AFL for the treatment of nAMD in Argentina, Colombia, Costa Rica, and Mexico, and anti-VEGF therapies are considered the gold standard for treatment of nAMD in Latin America. The recommended dose for treatment of nAMD with IVT-AFL is 2 mg (0.05 mL) administered monthly for the first 3 months, and then once every 2 months, for an approximate total of seven to eight injections during the first year [2].

Barriers to optimal patient care exist in Latin America and include patients not receiving the label-prescribed number of anti-VEGF injections, limited access to ophthalmology specialists, and lack of awareness of nAMD in general [1]. Inadequate health insurance may limit the number of injections a patient may receive and their access to ophthalmology specialists [5]; however, this varies by country.

In addition to these difficulties in optimizing patient care, significant risk factors for patient non-adherence and non-persistence to anti-VEGF treatment have been identified [6]. These risk factors further impair the achievement of efficacy outcomes from randomized controlled trials in routine clinical practice. Additional real-world evidence is required to fully understand the effectiveness of IVT-AFL in routine clinical practice, and could provide useful insights into current opportunities for clinical practice optimization.

AQUILA was a prospective observational cohort study in patients with nAMD or diabetic macular edema (DME), designed to Assess the freQuency of Use of IVT-AFL in routine clinical practice in Latin America (NCT03470103). The aim of this study was to evaluate the clinical effectiveness, safety, and treatment patterns of IVT-AFL in routine clinical practice in Latin America in patients with nAMD who are treatment-naïve or patients who previously received treatment (and switched to IVT-AFL).

## Methods

### Study design

The AQUILA study (NCT03470103) was conducted in accordance with the Declaration of Helsinki and the International Council for Harmonisation guideline E6: Good Clinical Practice. The protocol and any amendments were reviewed and approved by each study site’s Independent Ethics Committee or Institutional Review Board before the start of the study. AQUILA enrolled treatment-naïve and previously treated patients with nAMD (aged  ≥ 55 years) or DME (aged  ≥ 18 years) from March 2018, with a primary completion date of September 2020. Treatment-naïve patients had not previously received intravitreal treatment, including anti-VEGF agents, steroids, steroid implants, or photodynamic therapy. Previously treated patients had received different anti-VEGF therapy and were switching to IVT-AFL. Patients received IVT-AFL treatment at the discretion of the prescribing physician (according to their medical practice). The results for patients with DME are reported separately.

### Participants

Patients were enrolled from 13 clinics in Argentina, seven clinics in Colombia, two clinics in Costa Rica, and 11 clinics in Mexico. Patients became eligible for AQUILA once the decision was made to treat with IVT-AFL according to routine clinical practice (either receiving anti-VEGF therapy for the first time or switching from a different anti-VEGF therapy to IVT-AFL). Exclusion criteria included patients participating in a current clinical trial outside of routine practice; patients currently receiving IVT-AFL or another anti-VEGF agent for their disease; patients receiving a different anti-VEGF other than IVT-AFL in the fellow eye; patients receiving concomitant ocular or systemic administration drugs that could affect the mechanism of IVT-AFL; or patients with ocular or peri-ocular infections in either eye, or active intraocular inflammation, scar, fibrosis, atrophy, advanced glaucoma, or cataracts in the study eye.

### Study endpoints and analysis

The primary efficacy endpoint was change from baseline to month 12 in best-corrected visual acuity (BCVA; Early Treatment Diabetic Retinopathy Study [ETDRS] letters). Secondary endpoints included: treatment patterns at month 12 (number of injection/monitoring/combined visits, number of visual acuity [VA] tests, number of fundoscopy examinations, and number of optical coherence tomography [OCT] assessments); duration and type of previous treatments and reason for switch to IVT-AFL in previously treated patients; mean time between IVT-AFL injections and mean number of IVT-AFL injections at month 12; duration and type of previous treatments and reason for switch to IVT-AFL (previously treated subpopulation only); number of patients achieving a Snellen equivalent of 20/40 or better (~ 70 ETDRS letters) at month 12 and number of patients gaining  ≥ 15 ETDRS letters at month 12; change from baseline to month 12 in central retinal thickness (CRT); and number of patients with no fluid determined by OCT (absence of fluid includes all types of fluid, as determined by physician’s judgment) at month 12.

Patients who received at least one IVT-AFL injection were included in the safety analysis set (SAF). Patients were included in the full analysis set (FAS) if they received at least one IVT-AFL injection and had a BCVA assessment in the study eye at both baseline and at one or more follow-up visits. Data were analyzed descriptively. Last observation carried forward was used to impute missing values for BCVA and CRT measurements. Missing values for other variables, such as fluid, were not imputed.

## Results

### Baseline demographics and disease characteristics

Of the 327 patients screened for inclusion in this study, three did not receive treatment and were not included in the SAF. Of 324 patients in the SAF, 50 patients did not have a valid BCVA letter score at baseline or post-baseline and were ineligible for inclusion in the FAS; the overall FAS, therefore, comprised 274 patients (Additional file 1: Figure S1).

Patients were aged 55–96 years (mean 77 years), and 65% were female (Table 1). Comorbidities reported in  ≥ 5% of patients included hypertension (40.9%), cataracts (17.5%), hyperlipidemia (12.0%), and type 2 diabetes mellitus (8.0%). The mean BCVA letter score ± standard deviation (SD) in the study eye was 48.0 ± 22.9, and the mean CRT ± SD was 385 ± 137 µm. There were 201 treatment-naïve patients, and 73 patients were previously treated with ranibizumab (n = 36; 49%), bevacizumab (n = 41; 56%) (patients could have received both), or an unknown anti-VEGF agent (n = 1). The mean duration of previous treatments and the reasons for switching to IVT-AFL are shown in Additional file 1: Table S1.

**Table 1:** Patient baseline demographics and disease characteristics (FAS)

	Treatment-naïve (n = 201)	Previously treated (n = 73)	Overall (n = 274)
Age, years	77.6 ± 7.6	76.1 ± 8.3	77.2 ± 7.8
Female, n (%)	135 (67.2)	44 (60.3)	179 (65.3)
Country, n (%)			
Argentina	163 (81.1)	33 (45.2)	196 (71.5)
Colombia	12 (6.0)	7 (9.6)	19 (6.9)
Costa Rica	4 (2.0)	14 (19.2)	18 (6.6)
Mexico	22 (11.0)	19 (26.0)	41 (15.0)
BCVA in the study eye, letter score	48.2 ± 23.5	47.7 ± 21.4	48.0 ± 22.9
Categorical BCVA letter score, n (%)			
≥ 70 letters	42 (20.9)	14 (19.2)	56 (20.4)
< 70 letters	159 (79.1)	59 (80.8)	218 (79.6)
CRT, μm	378 ± 137	400 ± 137	385 ± 137

Values are mean ± SD unless otherwise stated

*BCVA* best-corrected visual acuity, *CRT* central retinal thickness, *FAS* full analysis set, *SD* standard deviation

In treatment-naïve patients, the median time from diagnosis of nAMD to first injection of IVT-AFL was 1.2 months (interquartile range [IQR]: 0.2–2.7); in previously treated patients who had already received a mean of 16.1 months of anti-VEGF treatment, the median time from diagnosis to first IVT-AFL injection was 19.5 months (IQR: 6.6–37.8).

### Treatment regimens and visits

The mean (± SD) number of IVT-AFL injections in treatment-naïve patients was 3.1 ± 0.9 by month 6 and 4.2 ± 1.9 by month 12; in previously treated patients, it was 3.6 ± 1.3 by month 6 and 5.2 ± 2.7 by month 12 (Table 2). A total of 170 of 274 patients (62%) received three or more initial monthly IVT-AFL injections by month 3 (127/201 treatment-naïve; 43/73 previously treated), and 47 of 274 patients (17%) received seven or more injections by month 12 (24/201 treatment-naïve; 23/73 previously treated) (Table 2). The mean dosing interval time (after the first 90 days) was 51.7 days (IQR: 37.0–61.5). Additional file 1: Table S2 contains the number of clinical visits for injections, monitoring visits without injections, and combined visits for injections and monitoring.

**Table 2:** Planned and actual dosing regimens and injections (FAS)

	Treatment-naïve (n = 201)	Previously treated (n = 73)	Overall (n = 274)
Planned dosing regimen			
T&E from initial treatment	51 (25.4)	8 (11.0)	59 (21.5)
3 initial monthly injections, then T&E	84 (41.8)	31 (42.5)	115 (42.0)
3 initial monthly injections, then every other month	5 (2.5)	4 (5.5)	9 (3.3)
Treat until dry, then T&E	12 (6.0)	19 (26.0)	31 (11.3)
Treat until dry, then PRN	20 (10.0)	2 (2.7)	22 (8.0)
PRN from initial treatment	17 (8.5)	6 (8.2)	23 (8.4)
Other	12 (6.0)	3 (4.1)	15 (5.5)
Reported dosing regimen^a^			
T&E from initial treatment	43 (21.4)	7 (9.6)	50 (18.3)
3 initial monthly injections, then T&E	40 (19.9)	25 (34.3)	65 (23.7)
3 initial monthly injections, then every other month	9 (4.5)	2 (2.7)	11 (4.0)
Treat until dry, then T&E	12 (6.0)	18 (24.7)	30 (11.0)
Treat until dry, then PRN	51 (25.4)	5 (6.9)	56 (20.4)
PRN from initial treatment	21 (10.5)	8 (11.0)	29 (10.6)
3 initial monthly injections not completed	7 (3.5)	4 (5.5)	11 (4.0)
Other	18 (9.0)	4 (5.5)	22 (8.0)
IVT-AFL injections by month 6 (mean ± SD)	3.1 ± 0.9	3.6 ± 1.3	3.2 ± 1.1
IVT-AFL injections by month 12 (mean ± SD)	4.2 ± 1.9	5.2 ± 2.7	4.4 ± 2.2
≥ 3 injections within 3 months	127 (63.2)	43 (58.9)	170 (62.0)
≥ 7 injections within 12 months	24 (11.9)	23 (31.5)	47 (17.2)

Data are n (%) unless otherwise stated

*FAS* full analysis set, *IVT-AFL* intravitreal aflibercept, *PRN pro re nata*, *SD* standard deviation, *T&E* treat and extend

^a^As reported by the investigator(s)

### Functional and anatomic outcomes

**Fig. 1:**
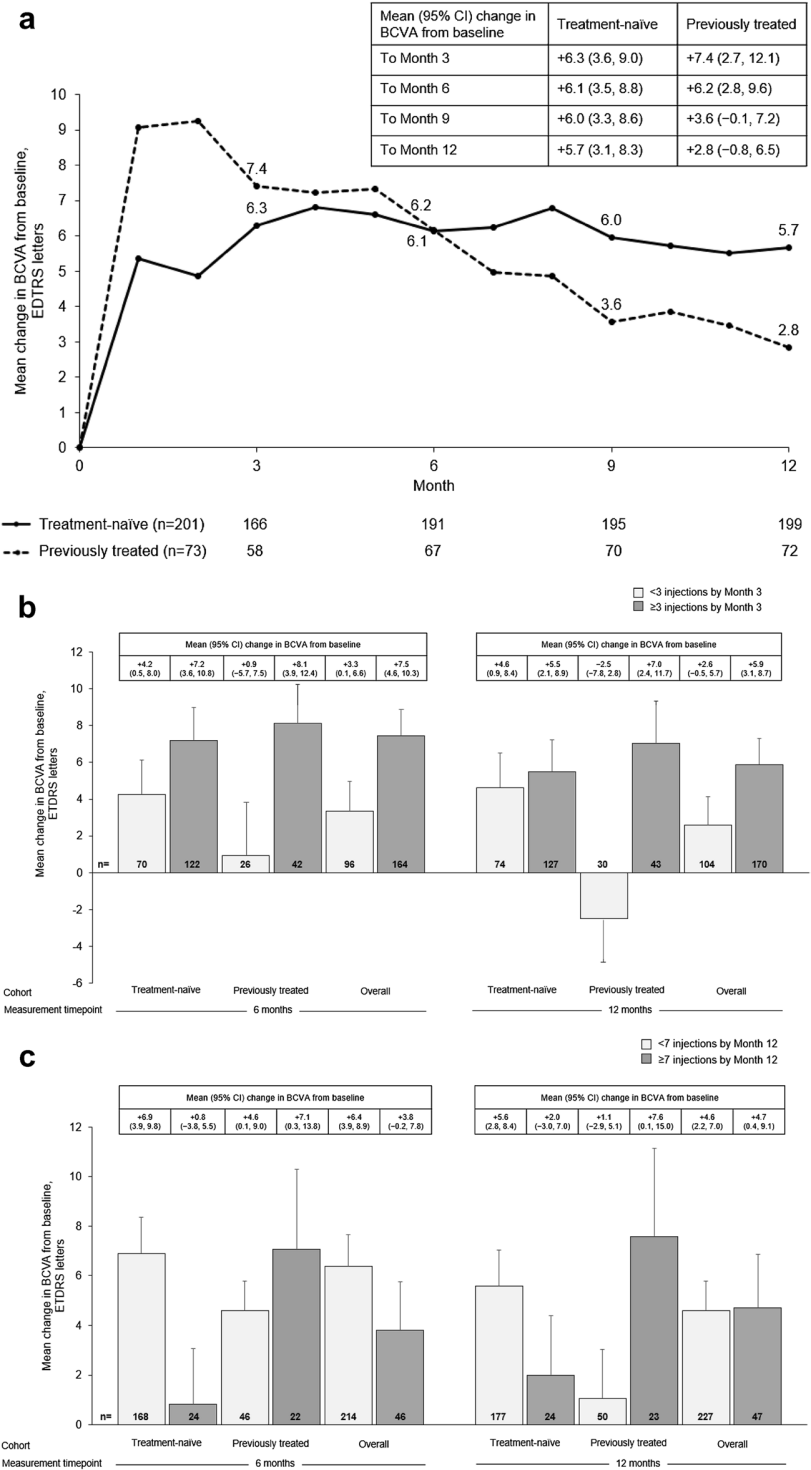

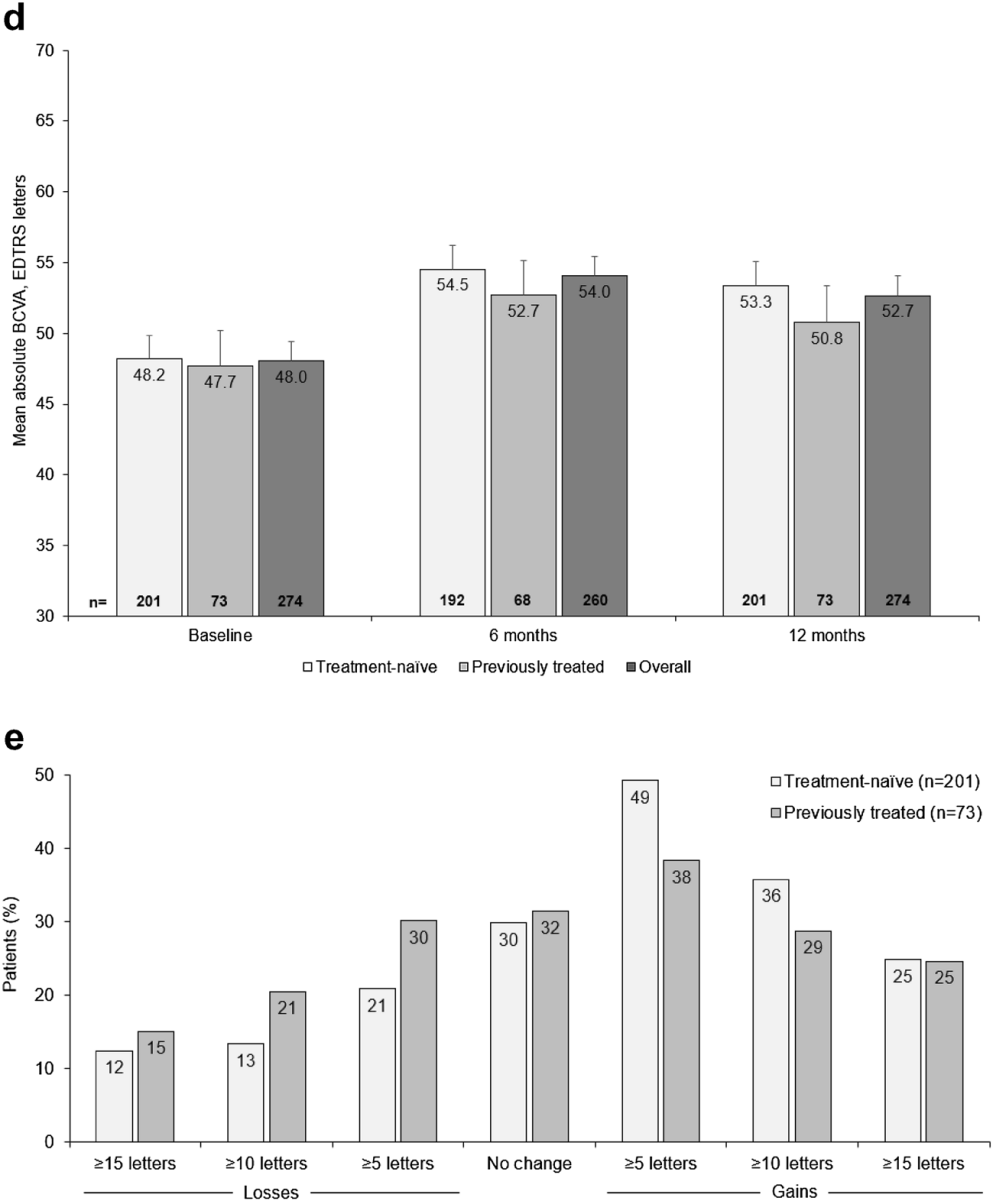
Visual acuity outcomes (FAS). **a**, Mean change in BCVA letter score over 12 months in treatment-naïve and previously treated patients; **b**, Mean change in BCVA letter score at months 6 and 12 in treatment-naïve and previously treated patients by number of injections received in the first 3 months of treatment; **c**, Mean change in BCVA letter score at months 6 and 12 in treatment-naïve and previously treated patients by overall number of injections; **d**, Mean absolute BCVA letter score at months 6 and 12 in treatment-naïve and previously treated patients; **e**, Proportion of treatment-naïve and previously treated patients by BCVA categorical score change at month 12. Missing data were imputed using LOCF. Data in **a** were collected monthly ± 15 days. In **b**–**d**, data for the 6-month time point were collected at 6 months ± 30 days; data for the 12-month time point were collected at 12 months ± 60 days. Error bars denote standard error. *BCVA* best-corrected visual acuity, *FAS* full analysis set, *LOCF* last observation carried forward

An improvement in BCVA over 12 months (the primary endpoint) was observed in both patient groups; the mean increase in BCVA at 12 months [95% CI] was numerically higher in treatment-naïve patients (+ 5.2 letters [2.6, 7.7]) than in previously treated patients (+ 3.1 letters [− 0.5, 6.7]). Mean change in BCVA over 12 months for treatment-naïve and previously treated patients is shown in Fig. 1A.

Patients who received three or more IVT-AFL injections in the initial phase of treatment (as per label recommendation [2]) had numerically higher gains in BCVA after 12 months of IVT-AFL treatment, regardless of prior treatment (Fig. 1B). BCVA gains were similar among cohorts at 6 months and 12 months when stratifying by number of injections received over the course of AQUILA (Fig. 1C). Figure 1D depicts mean absolute BCVA letter score; gains were numerically higher in treatment-naïve patients. By month 12, 25% of patients in both groups had BCVA improvements of  ≥ 15 letters (Fig. 1E), and the proportion of patients with a loss of  ≥ 15 letters by month 12 was 12.4% in the treatment-naïve cohort, and 15.1% in the previously treated cohort. The proportion of patients with BCVA  ≥ 70 letters increased from 20.9% at baseline to 37.3% at month 12 in treatment-naïve patients and from 19.2% at baseline to 31.5% at month 12 in previously treated patients.

**Fig. 2:**
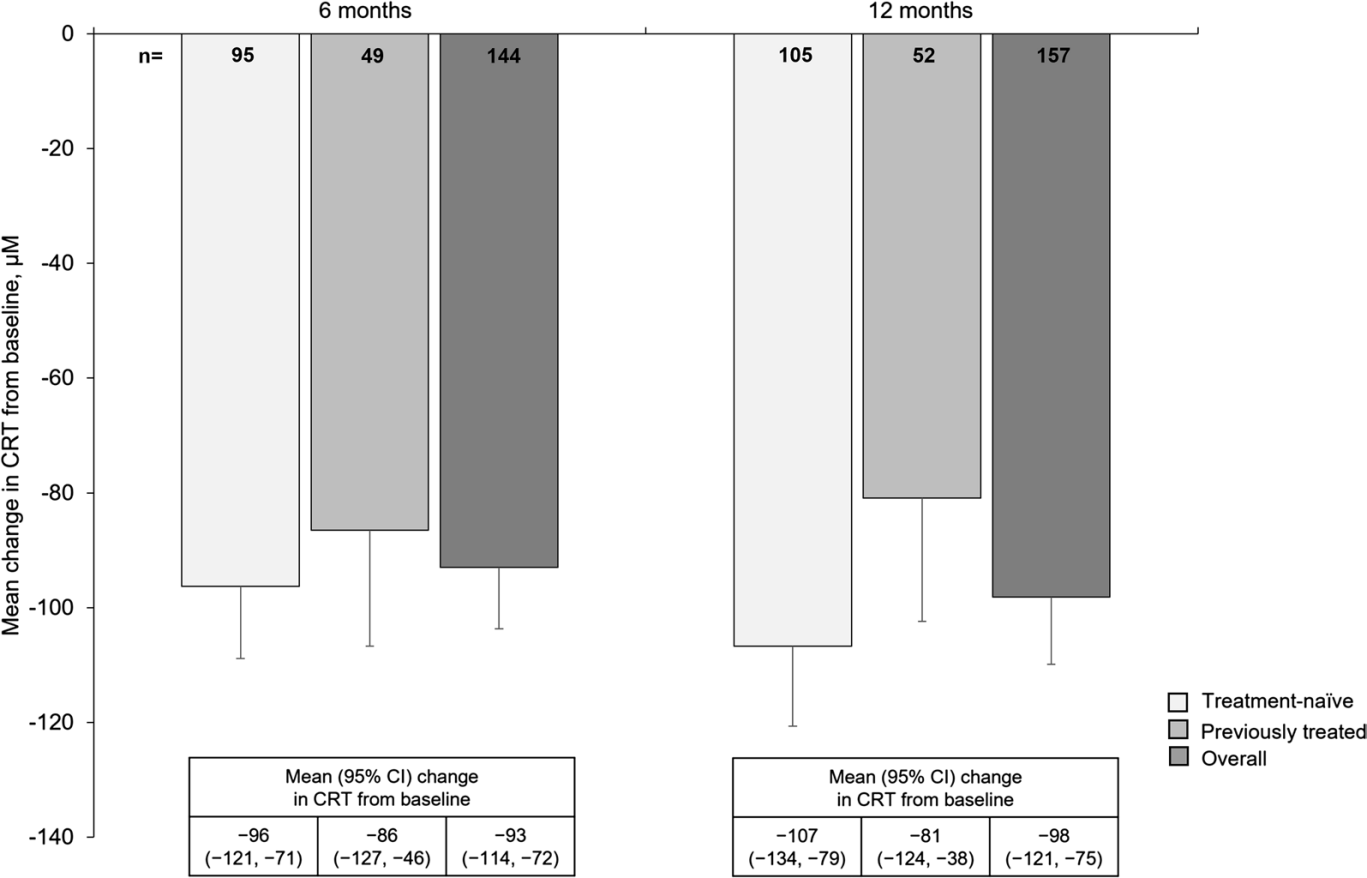
Mean change in CRT over 12 months in treatment-naïve and previously treated patients (FAS). Missing data were imputed using LOCF. Error bars denote standard error. *CRT* central retinal thickness, *FAS* full analysis set, *LOCF* last observation carried forward

By month 12, mean CRT (mean ± SD) decreased by 107 ± 143 μm (treatment-naive; from baseline of 378 ± 137) and 81 ± 155 μm (previously treated; from baseline of 400 ± 137) (Fig. 2).

The proportion of patients with intraretinal, subretinal, and subretinal pigment epithelium fluid at baseline and after 12 months’ treatment with IVT-AFL are shown in Additional file 1: Figure S2; the proportion of patients without any fluid increased from 3.7% at baseline to 35% at month 12. A total of 28 patients were unable to complete 12 months due to COVID-19, and the mean change in BCVA up to 12 months was + 4.8 letters in patients treated pre–COVID-19 (n = 224), and + 3.9 letters in patients treated during the pandemic (n = 50).

### Safety

An overview of the main safety data is shown in Table 3. Ocular adverse events (AEs) were reported in 5.6% (n = 18) of patients; the most common were cataract (0.9%, n = 3) and conjunctival hemorrhage (0.9%, n = 3). There were no cases of endophthalmitis, retinal vasculitis, or retinal artery occlusion. Five treatment-related AEs were reported; all were ocular-related (two incidences of conjunctival hemorrhage and one incidence each of iridocyclitis, ocular hypertension, and increase in intraocular pressure). Serious ocular AEs occurring in seven patients were cataract, conjunctival hemorrhage, worsening of macular degeneration, ocular hypertension, retinal detachment, retinal hemorrhage, rhegmatogenous retinal detachment, and vitreous hemorrhage (one incidence each); the conjunctival hemorrhage and ocular hypertension were considered treatment-related by the treating physician. Serious non-ocular AEs were reported by two patients: injuries from a road traffic accident, and an aortic valve replacement. Two deaths were reported during the 12-month study; neither were treatment related.

**Table 3:** Safety overview

Patients, n (%)	Safety analysis set (N = 324)
Any AE^a^	24 (7.4)
Ocular AEs^b^	18 (5.6)
Cataract	3 (0.9)
Conjunctival hemorrhage	3 (0.9)
Treatment-related ocular AEs	5 (1.5)
Serious ocular AEs	7 (2.2)
Treatment-related serious ocular AEs	2 (0.6)
Non-ocular AEs	6 (1.9)
Treatment-related non-ocular AEs	0
Serious non-ocular AEs	2 (0.6)
Deaths^c^	2 (0.6)

^a^AEs are those reported if they started after the first IVT-AFL injection and not later than 30 days after the last IVT-AFL injection. If no unambiguous allocation is possible because of missing parts of the AE start date for example, the AE will be treated as an AE (worst case scenario)

^b^Ocular AEs reported by preferred term in  ≥ 3 patients

^c^One patient died of myocardial infarction, and 1 patient died of prostate cancer

*AE* adverse event, *IVT-AFL* intravitreal aflibercept

## Discussion

AQUILA is one of the first observational, real-world studies of anti-VEGF agents in Latin America, and the first to assess the use of IVT-AFL in routine clinical practice in Latin America. Patients with nAMD who received IVT-AFL at the direction of the treating physician had improved functional and anatomic outcomes after 12 months of treatment, regardless of previous treatment status. Improvements in BCVA were numerically greater at 12 months in treatment-naïve patients (+ 5.2 letters) than in previously treated patients (+ 3.1 letters). CRT decreased from baseline to month 12 by 107 μm in treatment-naïve patients and by 81 μm in previously treated patients. Despite many patients in AQUILA not receiving the label-recommended number of injections, visual outcomes still showed improvement following 12 months of treatment, although not to the extent observed in randomized controlled trials. Patients who received three or more initial monthly injections had numerically greater improvements in BCVA than those who received fewer than three initial monthly injections. No new safety concerns were observed.

VIEW 1 and VIEW 2 were two similarly designed, global, phase 3 studies of IVT-AFL in treatment-naïve patients with nAMD [4]. VIEW 1/2 were designed to assess IVT-AFL treatment noninferiority to ranibizumab; patients received IVT-AFL treatment for 12 months in one of three fixed dosing regimens (2q4, 0.5q4, and 2q8 [after three initial monthly injections]) or they received 0.5 mg ranibizumab monthly). Patients receiving anti-VEGF treatment according to their regimen achieved similar gains in BCVA (+ 9.3, + 8.3, + 8.4, and + 8.7 letters in patients receiving 2q4, 0.5q4, 2q8, and ranibizumab, respectively) by month 12. Patients in AQUILA, treated outside of the strictly controlled clinical trial environment, received fewer injections than patients in VIEW 1 and VIEW 2 (4.4 ± 2.2 injections by month 12 overall). Visual gains were clinically relevant, though lower than those achieved in VIEW 1 and 2. The AQUILA visual outcomes were consistent with previous observational studies of IVT-AFL (and indeed other anti-VEGF agents) treatment in routine clinical practice and have highlighted the disparity between label-recommended number of doses in clinical trials, and number of doses received during routine clinical practice [7–9].

LUMINOUS was a global, prospective, observational study of ranibizumab in patients with nAMD (and also for patients with DME or retinal vein occlusion), providing real-world evidence of the effectiveness of anti-VEGF treatment for nAMD [10, 11]. LUMINOUS enrolled treatment-naïve (n = 6241) and previously treated (n = 16 167) patients globally (including from Argentina, Colombia, Costa Rica, and Mexico [10, 11]). Baseline BCVA was 49.7 letters in LUMINOUS treatment-naïve patients compared with 48.2 letters in AQUILA treatment-naïve patients, and 58.3 letters in LUMINOUS previously treated patients compared with 47.7 in patients who previously received IVT-AFL in AQUILA [10, 11]. The baseline BCVA of previously treated patients in AQUILA is low when compared with other observational anti-VEGF studies including a previously treated patient arm [7, 11]. In AQUILA, the median time between diagnosis and first injection of IVT-AFL was 1.2 months in treatment-naïve patients, compared to 19.5 months in patients who received previous treatment; taken together, this may indicate a lack of efficacy of patient’s previous therapy (therefore their physician switched the patient’s therapy to IVT-AFL, allowing the patient to enroll in AQUILA; Additional file 1: Table S1), and therefore a comparably low baseline BCVA. Treatment-naïve patients (n = 3379) gained + 3.1 letters with an average of 5.0 ranibizumab injections up to month 12 of LUMINOUS [10]. Despite treatment-naïve patients in AQUILA receiving fewer IVT-AFL injections (4.2 injections over 12 months), gains in BCVA were numerically higher (+ 5.2 letters). Patients who previously received therapy for nAMD in LUMINOUS lost an average of 1.6 letters after 1 year of ranibizumab treatment; LUMINOUS patients who received more frequent injections over 12 months had more positive visual outcomes. In comparison, previously treated patients in AQUILA gained 3.1 letters after 12 months of IVT-AFL treatment. However, previously treated patients in AQUILA had a lower mean BCVA at baseline than patients in LUMINOUS, and patients in AQUILA received a numerically higher number of injections than those enrolled in LUMINOUS (5.2 in AQUILA vs 4.7 in LUMINOUS) [11].

The Pan-American Collaborative Retina Study Group (PACORES) investigated 1-year outcomes following bevacizumab treatment for primary choroidal neovascularization secondary to nAMD in 60 patient eyes [12]. The number of injections received by patients in the trial was fewer than in those enrolled in AQUILA, with similar visual outcomes. Despite most patients in AQUILA not receiving the label-prescribed number of injections, many patients achieved improvements in both functional and anatomic outcomes, although not of the magnitude reported in prior clinical trials [4]. This suggests that if patients received the label-prescribed number of injections during AQUILA, they may have achieved larger gains in visual outcomes. This is consistent with data from longer-term studies suggesting that more frequent injections during the first year of treatment result in higher gains in VA [13].

In AQUILA, > 60% of patients with nAMD received three or more initial monthly doses of IVT-AFL, but  < 20% received seven or more injections in the first year of treatment. Despite the intention to treat according to the label, many patients were treated reactively over the course of AQUILA. The decision to treat reactively may be linked to issues of treatment reimbursement. Treatment reimbursement in Latin America varies by country. In Argentina, anti-VEGF reimbursement depends on the payer, and patients pay for the treatment that they can afford. In Colombia, treatment costs are covered by self-paid insurance. Costa Rica’s national health care system is funded by taxpayers through employment taxes, with contributions from both the payer and employer; however, bevacizumab is the only anti-VEGF agent available via the national health care system. Patients from Costa Rica and Mexico who enrolled in AQUILA paid out of pocket for their treatment; these costs were reimbursed if patients had private health insurance. Indeed, the AQUILA data should be taken in context with treatment availability as dictated by the different Latin American health care systems. As over 70% of patients in the nAMD cohort of AQUILA come from Argentina, the Argentinian health care system largely influences the treatment pattern data in this study.

The safety profile of IVT-AFL was consistent with that of previous clinical and observational studies [4, 7, 14, 15]. No incidences of endophthalmitis were reported.

Reliance on BCVA as the key efficacy parameter is one limitation of this study; furthermore the data in this study may not give the full picture of treatment patterns and the landscape within Latin America, as it does not include Brazil, the country with the largest population in this region. A large proportion of CRT and fluid data are missing at month 12; this could be due to country-specific reimbursement for OCT testing, limiting the number of patients willing to pay for the examination, or due to availability of fluid measurement equipment. Furthermore, the BCVA results observed for patients with a certain number of IVT-AFL injections, and those without, were determined post-baseline and post hoc, and any interpretation must therefore consider their relation as associative, rather than causative.

## Conclusions

To conclude, AQUILA is the first study to assess the use of IVT-AFL in routine clinical practice in Latin America. In AQUILA, despite few patients receiving the recommended regimen of  ≥ 7 IVT-AFL injections in the first year of treatment, functional and anatomic outcomes improved during 12 months of treatment. Improvements in BCVA were numerically greater in treatment-naïve patients than in previously treated patients, and in patients who received  ≥ 3 initial monthly injections than in those who did not. Thus, in real-world studies, patients with nAMD treated regularly and more frequently with IVT-AFL have the potential to achieve outcomes consistent with outcomes observed in interventional studies.

## Supplementary Information

**Additional file 1: Table S1.** Duration of previous treatment for nAMD (previously treated, FAS) and reasons for switch to IVT-AFL. **Table S2.** Proportion of patients with 0, 1–3, 4–6, 7–9, and ≥10 clinical, monitoring, or combined visits by Month 12 and proportion of patients with a non-ophthalmology visit by Month 12 (FAS). **Figure S1.** Patient disposition. **Figure S2.** Fluid status at (a) baseline, (b) Month 6, and (c) Month 12 in treatment-naïve and previously treated patients.

## Data Availability

Availability of the data underlying this publication will be determined according to Bayer’s commitment to the EFPIA/PhRMA “Principles for responsible clinical trial data sharing”. This pertains to scope, timepoint, and process of data access. As such, Bayer commits to sharing upon request from qualified scientific and medical researchers patient-level clinical trial data, study-level clinical trial data, and protocols from clinical trials in patients for medicines and indications approved in the United States (US) and European Union (EU) as necessary for conducting legitimate research. This applies to data on new medicines and indications that have been approved by the EU and US regulatory agencies on or after January 01, 2014. Interested researchers can use www.clinicalstudydatarequest.com to request access to anonymized patient-level data and supporting documents from clinical studies to conduct further research that can help to advance medical science or improve patient care. Information on the Bayer criteria for listing studies and other relevant information is provided in the ‘Study sponsors’ section of the portal. Data access will be granted to anonymized patient-level data, protocols, and clinical study reports after approval by an independent scientific review panel. Bayer is not involved in the decisions made by the independent review panel. Bayer will take all necessary measures to ensure that patient privacy is safeguarded.

## Abbreviations

AE: Adverse event
Anti-VEGF: Anti-vascular endothelial growth factor
BCVA: Best-corrected visual acuity
CRT: Central retinal thickness
DME: Diabetic macular edema
ETDRS: Early Treatment Diabetic Retinopathy Study
FAS: Full analysis set
IQR: Interquartile range
IVT-AFL: Intravitreal aflibercept
LOCF: Last observation carried forward
nAMD: Neovascular age-related macular degeneration
OCT: Optical coherence tomography
PRN: PRN, pro re nata
SAF: Safety analysis set
T&E: Treat and extend
VA: Visual acuity
VEGF: Vascular endothelial growth factor

## References

[1] Pan-American retina and vitreous society and the angiogenesis foundation. Advocating for improved treatment and outcomes for wet age-related macular degeneration. 2012. https://Angio.Org/Wp-Content/Uploads/2013/10/Latin_America_Amd_Expert_Summit_White_Paper-English.Pdf. Accessed May 2022.

[2] Eylea [Prescribing Information]. Tarrytown: Regeneron Pharmaceuticals, Inc.; 2021. https://www.regeneron.com/downloads/eylea_fpi.pdf. Accessed Aug 2022.

[3] Trichonas G, Kaiser PK (2013). Aflibercept for the treatment of age-related macular degeneration. Ophthalmol Ther.

[4] Heier JS, Brown DM, Chong V, Korobelnik JF, Kaiser PK, Nguyen QD (2012). Intravitreal aflibercept (VEGF trap-eye) in wet age-related macular degeneration. Ophthalmology.

[5] Hong H, Mujica OJ, Anaya J, Lansingh VC, Lopez E, Silva JC (2016). The challenge of universal eye health in Latin America: distributive inequality of ophthalmologists in 14 countries. BMJ Open.

[6] Okada M, Mitchell P, Finger RP, Eldem B, Talks SJ, Hirst C (2020). Non-adherence or non-persistence to intravitreal injection therapy for neovascular age-related macular degeneration: a mixed-methods systematic review. Ophthalmology.

[7] Framme C, Eter N, Hamacher T, Hasanbasic Z, Jochmann C, Johnson KT (2018). Aflibercept for patients with neovascular age-related macular degeneration in routine clinical practice in Germany: twelve-month outcomes of PERSEUS. Ophthalmol Retina.

[8] Kim LN, Mehta H, Barthelmes D, Nguyen V, Gillies MC (2016). Metaanalysis of real-world outcomes of intravitreal ranibizumab for the treatment of neovascular age-related macular degeneration. Retina (Philadelphia, Pa).

[9] Weber M, Dominguez M, Coscas F, Faure C, Baillif S, Kodjikian L, Cohen SY (2020). Impact of intravitreal aflibercept dosing regimens in treatment-naïve patients with neovascular age-related macular degeneration: 2-year results of RAINBOW. Bmc Ophthalmol.

[10] Holz FG, Figueroa MS, Bandello F, Yang Y, Ohji M, Dai H (2020). Ranibizumab treatment in treatment-naive neovascular age-related macular degeneration: results from LUMINOUS, a global real-world study. Retina (Philadelphia, Pa).

[11] Holz FG, Minnella AM, Tuli R, Yoganathan P, Parikh S, Hamilton R (2020). Ranibizumab treatment patterns in prior ranibizumab-treated neovascular age-related macular degeneration patients: real-world outcomes from the LUMINOUS study. PLoS One.

[12] Wu L, Fernando Arevalo J, Maia M, Berrocal Mh, Sanchez J, Evans T (2009). Comparing outcomes in patients with subfoveal choroidal neovascularization secondary to age-related macular degeneration treated with two different doses of primary intravitreal bevacizumab: results of the Pan-American Collaborative Retina Study Group (PACORES) at the 12-month follow-up. Jpn J Ophthalmol.

[13] Almuhtaseb H, Johnston RL, Talks JS, Lotery AJ (2017). Second-year visual acuity outcomes of nAMD patients treated with aflibercept: data analysis from the UK Aflibercept Users Group. Eye (Lond).

[14] Carrasco JDV, Eldem BM, Spoorendonk JA, Yoon J (2021). 2-year real-world outcomes with intravitreal aflibercept in neovascular age-related macular degeneration: systematic review and meta-analysis of patient-relevant outcomes. Ophthalmol Ther.

[15] Weber M, Velasque L, Coscas F, Faure C, Aubry I, Cohen SY (2019). Effectiveness and safety of intravitreal aflibercept in patients with wet age-related macular degeneration treated in routine clinical practices across France: 12-month outcomes of the RAINBOW study. BMJ Open Ophthalmol.

