# Supplementary material for: Intravitreal aflibercept for the treatment of patients with neovascular age-related macular degeneration in routine clinical practice in Latin America: the AQUILA study
Source: Int J Retina Vitreous. 2022 Oct 18;8:76. doi: 10.1186/s40942-022-00425-w (PMC9579549; doi:10.1186/s40942-022-00425-w)
Supplement: Supplementary file 1 — Additional file 1: Table S1. Duration of previous treatment for nAMD (previously treated, FAS) and reasons for switch to IVT-AFL. Table S2. Proportion of patients with 0, 1–3, 4–6, 7–9, and ≥10 clinical, monitoring, or combined visits by Month 12 and proportion of patients with a non-ophthalmology visit by Month 12 (FAS). Figure S1. Patient disposition. Figure S2. Fluid status at (a) baseline, (b) Month 6, and (c) Month 12 in treatment-naïve and previously treated patients. [file 40942_2022_425_MOESM1_ESM.docx]

**Supplementary content**

**23 May 2022**

# Title:

Intravitreal aflibercept for the treatment of patients with neovascular age-related macular degeneration in routine clinical practice in Latin America: the AQUILA study

**Authors:**

Lihteh Wu,^1^ Arnaldo F. Bordon,^2^ Martin Charles,^3^ Francisco J. Rodríguez,^4^ JinKyung Lee,^5^ Tobias Machewitz,^5^ Margarete Mueller,^5^ Gabriela del Carmen Gay,^6^ Jans Fromow-Guerra,^7^ on behalf of the AQUILA investigators

**Affiliations:**^1^Asociados de Mácula Vitreo y Retina de Costa Rica, San José, Costa Rica
^2^Hospital Oftalmológico de Sorocaba, Sorocaba, Brazil
^3^Centro Oftalmológico Dr Charles, Buenos Aires, Argentina
^4^Fundación Oftalmológica Nacional, Universidad del Rosario School of Medicine, Bogotá, Colombia
^5^Bayer AG, Berlin, Germany
^6^Bayer SA, Munro, Argentina
^7^Macula Retina Consultores, Mexico City, Mexico

**Correspondence to:**

**Lihteh Wu**

Asociados de Mácula Vitreo y Retina de Costa Rica
Primer Piso Torre Mercedes,
Calle 24, Paseo Colón,
San José 10102,
Costa Rica

[lihteh@gmail.com](mailto:lihteh@gmail.com)

Table of Contents

[1](#_Toc98501589)

[**Supplementary Table 1.** Duration of previous treatment for nAMD (previously treated, FAS) and reasons for switch to IVT-AFL **3**](#_Toc98501590)

[**Supplementary Table 2.** Proportion of patients with 0, 1–3, 4–6, 7–9, and ≥10 clinical, monitoring, or combined visits by month 12 and proportion of patients with a non-ophthalmology visit by month 12 (FAS) **4**](#_Toc98501591)

[**Supplementary Fig. 1.** Patient disposition **5**](#_Toc98501592)

[**Supplementary Fig. 2.** Fluid status at (**a**) baseline, (**b**) Month 6, and (**c**) Month 12 in treatment-naïve and previously treated patients **6**](#_Toc98501593)

[**Appendix.** List of participating investigators and clinics**. 7**](#_Toc98501594)

# Supplementary Table 1. Duration of previous treatment for nAMD (previously treated, FAS) and reasons for switch to IVT-AFL

| **Time between first and last treatments in months, mean ± SD^a^** |  |
| --- | --- |
| Ranibizumab (n = 34) | 11.2 ± 18.0 |
| Bevacizumab (n = 38) | 18.3 ± 26.1 |
| Total (n = 67) | 16.1 ± 23.7 |
| **Primary reason to switch to IVT-AFL, n (%)** |  |
| Persistent fluid (intraretinal or subretinal) | 42 (57.5) |
| Recurrence of fluid | 7 (9.6) |
| Decreased vision | 6 (8.2) |
| Effort to extend treatment interval | 5 (6.9) |
| Patient request | 5 (6.9) |
| New hemorrhage – bleed | 2 (2.7) |
| Lack of compliance | 2 (2.7) |
| Other | 4 (5.5) |

^a^Missing n = 6. FAS, full analysis set; IVT-AFL, intravitreal aflibercept; nAMD, neovascular age-related macular degeneration; SD, standard deviation.

# Supplementary Table 2. Proportion of patients with 0, 1–3, 4–6, 7–9, and ≥10 clinical, monitoring, or combined visits by Month 12 and proportion of patients with a non-ophthalmology visit by Month 12 (FAS)

|  | **n (%)** | **Treatment-naïve (n = 201)** | **Previously treated (n = 73)** | **Overall (n = 274)** |  |
| --- | --- | --- | --- | --- | --- |
| Clinical  visits^a^ | None | 40 (19.9) | 29 (39.7) | 69 (25.2) |  |
|  | 1–3 | 132 (65.7) | 33 (45.2) | 165 (60.2) |  |
|  | 4–6 | 28 (13.9) | 8 (11.0) | 36 (13.1) |  |
|  | 7–9 | 1 (0.5) | 3 (4.1) | 4 (1.5) |  |
|  | ≥10 | 0 | 0 | 0 |  |
| Monitoring visits^b^ | | None | 9 (4.5) | 13 (17.8) | 22 (8.0) |
|  |  | 1–3 | 73 (36.3) | 33 (45.2) | 106 (38.7) |
|  |  | 4–6 | 63 (31.3) | 13 (17.8) | 76 (27.7) |
|  |  | 7–9 | 36 (17.9) | 11 (15.1) | 47 (17.2) |
|  |  | ≥10 | 20 (10.0) | 3 (4.1) | 23 (8.4) |
| Combined  visits^c^ | | 1–3 | 170 (84.6) | 48 (65.8) | 218 (79.6) |
|  |  | 4–6 | 19 (9.5) | 11 (15.1) | 30 (11.0) |
|  |  | 7–9 | 11 (5.5) | 9 (12.3) | 20 (7.3) |
|  |  | ≥10 | 1 (0.5) | 5 (6.9) | 6 (2.2) |

^a^Clinical visit for injection. ^b^Visit only for diagnostic purposes, without injections. ^c^Visit for monitoring and injections. FAS, full analysis set.

# Supplementary Fig. 1. Patient disposition


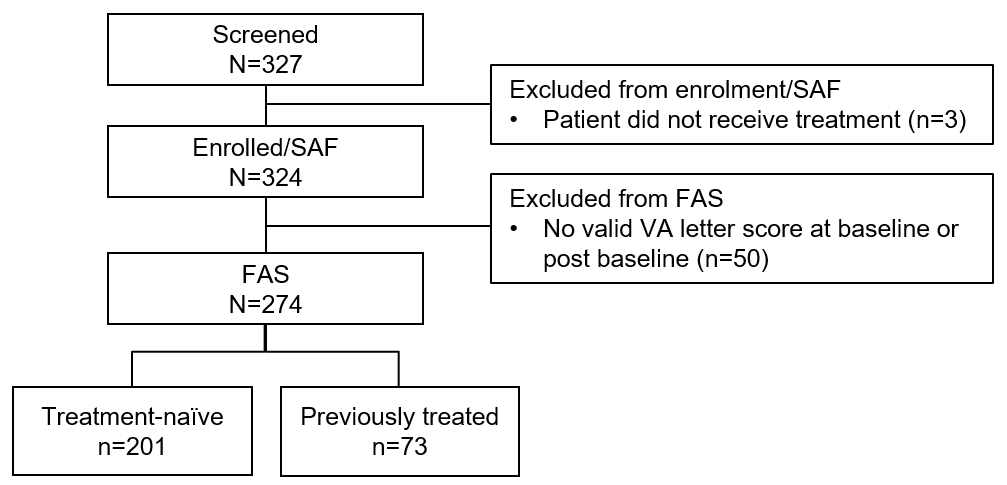


313 patients completed up to 6 months of treatment, and 215 patients completed 12 months of treatment. FAS, full analysis set; SAF, safety analysis set; VA, visual acuity.

# Supplementary Fig. 2. Fluid status at (a) baseline, (b) Month 6, and (c) Month 12 in treatment-naïve and previously treated patients

**a**


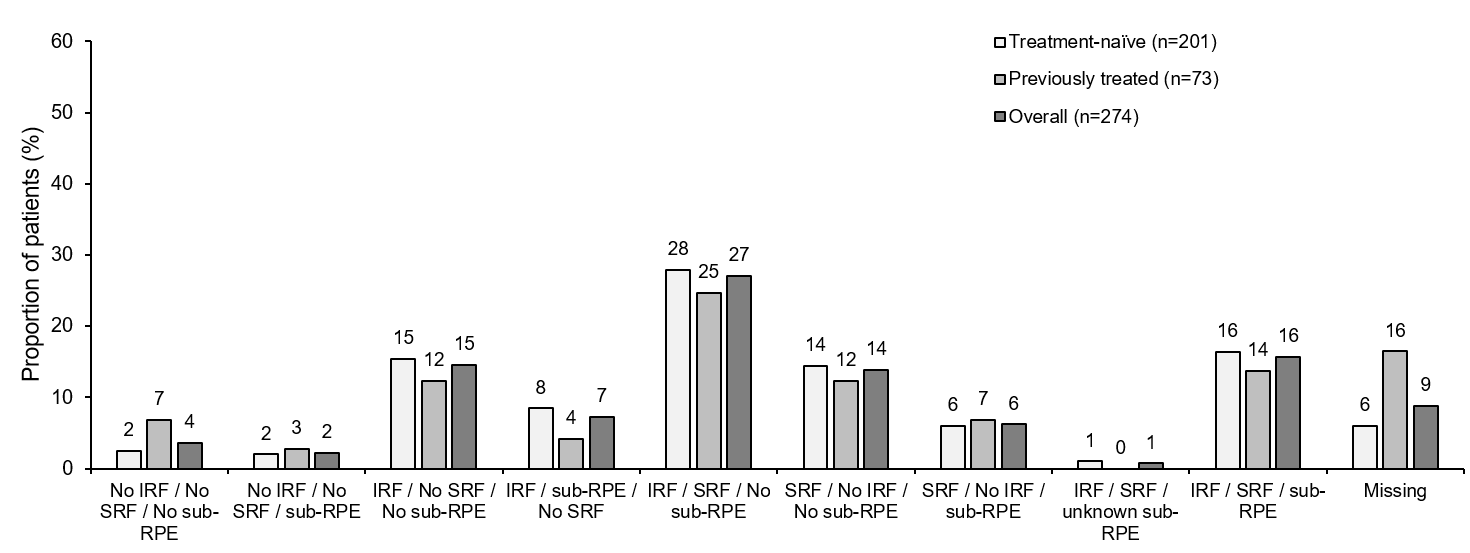


**b**

**
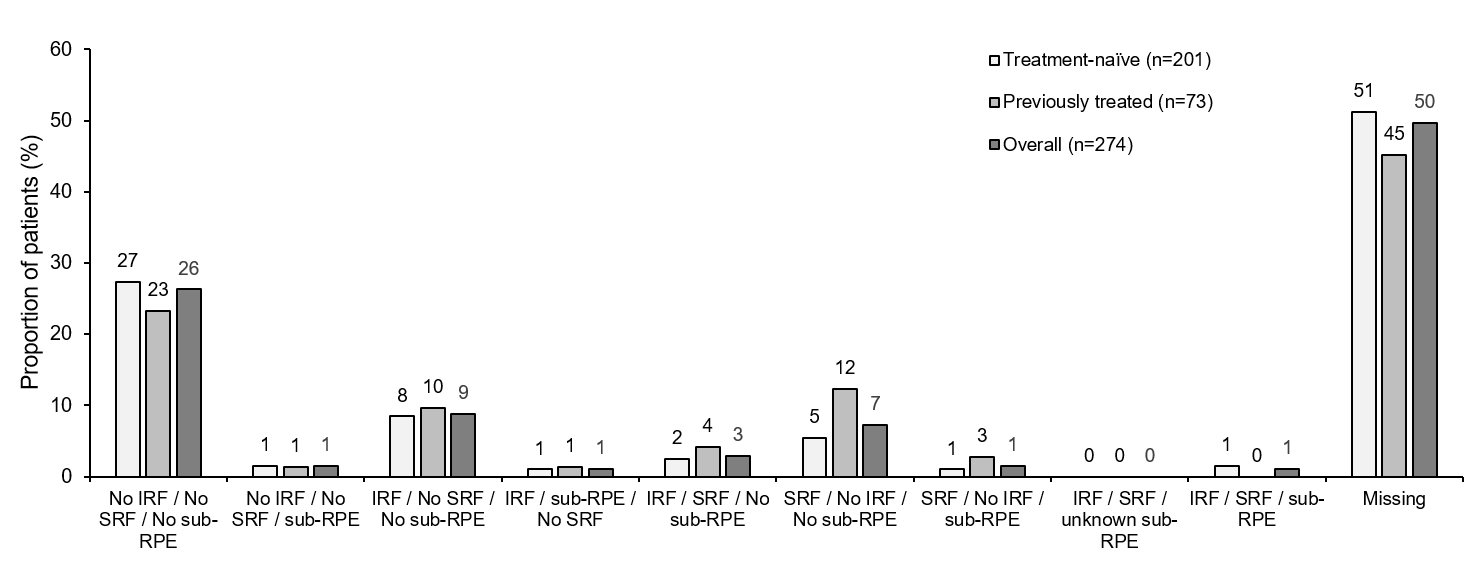
**

**c**

**
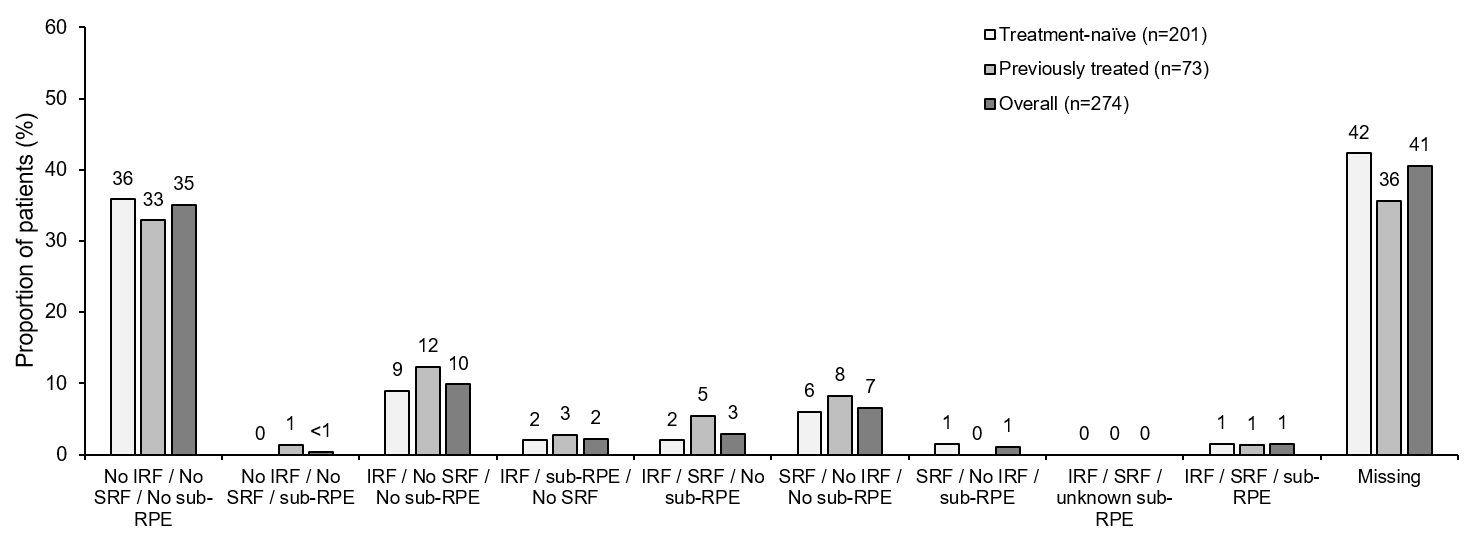
**

IRF, intraretinal fluid; RPE, retinal pigment epithelium; SRF, subretinal fluid.

# Appendix. List of participating investigators and clinics.

**Argentina:** Marcelo Reinhart (Olavarría); Gastón Gómez Caride (Quilmes); Herminio Negri (Ciudad Autonoma de Buenos Aires); Gerónimo Galván (La Plata, Buenos Aires); Juan Irungaray (Lanus Oeste); Mariano Irós (Córdoba); Matko Vidosevich (Rosario); Noe Rivero (Villa Gdor. Galvez); Tamara Zompa (Ciudad Autonoma de Buenos Aires); Juan Pablo Francos (Ciudad Autonoma de Buenos Aires); Paula Salgado (Ciudad Autonoma de Buenos Aires); Gerardo Caceres Barrios (Escobar); Octavio Regnasco (Escobar); **Colombia**: Francisco J. Rodríguez (Bogotá); Hildegard Piñeros (Barranquilla); Juan Arias (Bucaramanga); Javier Buendia (Medellín); Gustavo Adolfo Navarro Naranjo (Popayan); Beatriz Endo (Cali); Myrian Hernandez (Manizales); **Costa Rica:** Lihteh Wu (San José), Teodoro Evans Tinoco (San José); **Mexico:** Gerardo Garcia (Ciudad de México); Andres Padilla (Guadalajara); Adriana Gómez Cespedes (Chihuahua); Jose Dalma (Ciudad de México); Rene Cano (Ciudad de México); Jans Fromow-Guerra (Ciudad de México); Natalia Saldaña (Ciudad de México); Juan Manuel Jimenez (Ciudad de México); Renata del Carmen García Franco (Queretaro); Adriana Solis Vivanco (Mexico D.F.); Angeles Yael Hernandez Vazquez (Cuatitlan Izcalli).
